# Twenty Years of Progress Toward West Nile Virus Vaccine Development

**DOI:** 10.3390/v11090823

**Published:** 2019-09-05

**Authors:** Jaclyn A. Kaiser, Alan D.T. Barrett

**Affiliations:** 1 Department of Microbiology and Immunology, University of Texas Medical Branch, Galveston, TX 77555, USA; 2 Department of Pathology, University of Texas Medical Branch, Galveston, TX 77555, USA; 3 Sealy Institute for Vaccine Sciences, University of Texas Medical Branch, Galveston, TX 77555, USA

**Keywords:** West Nile virus, vaccine, flavivirus

## Abstract

Although West Nile virus (WNV) has been a prominent mosquito-transmitted infection in North America for twenty years, no human vaccine has been licensed. With a cumulative number of 24,714 neurological disease cases and 2314 deaths in the U.S. since 1999, plus a large outbreak in Europe in 2018 involving over 2000 human cases in 15 countries, a vaccine is essential to prevent continued morbidity, mortality, and economic burden. Currently, four veterinary vaccines are licensed, and six vaccines have progressed into clinical trials in humans. All four veterinary vaccines require multiple primary doses and annual boosters, but for a human vaccine to be protective and cost effective in the most vulnerable older age population, it is ideal that the vaccine be strongly immunogenic with only a single dose and without subsequent annual boosters. Of six human vaccine candidates, the two live, attenuated vaccines were the only ones that elicited strong immunity after a single dose. As none of these candidates have yet progressed beyond phase II clinical trials, development of new candidate vaccines and improvement of vaccination strategies remains an important area of research.

## 1. West Nile Virus Background

West Nile virus (WNV) is a member of the flavivirus genus and is related to other medically relevant mosquito-borne flaviviruses including dengue (DENV), yellow fever (YFV), Zika (ZIKV), and Japanese encephalitis (JEV) [1]. The flavivirus genome is comprised of positive-sense RNA approximately 11 kilobases in length encoding a single polyprotein that is cleaved into ten viral proteins: the structural proteins capsid (C), pre-membrane (prM), envelope (E), as well as the nonstructural proteins NS1, NS2A, NS2B, NS3, NS4A, NS4B, and NS5.

First isolated in Africa in 1937, WNV is now broadly distributed across the world [2,3]. It has a transmission cycle involving birds and *Culex spp*. mosquitoes and is the most common mosquito-borne disease in the United States [4]. Although it has been endemic in the U.S. for twenty years and there are veterinary vaccines available, there is no vaccine licensed for human use. 

**Figure 1:**
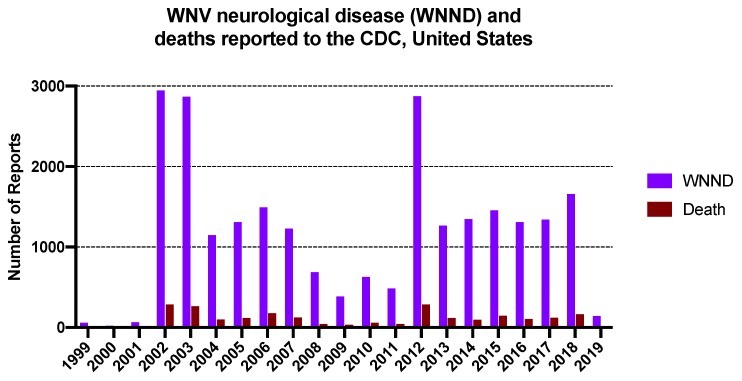
Annual morbidity and mortality associated with WNV in the United States. WNV neurological disease cases (purple bars) and deaths (red bars) that were reported to the United States Centers for Disease Control and Prevention are displayed. As of August 26, 2019, 143 cases of WNND and 11 fatalities have been reported for the year.

Human WNV infections can be categorized into a typical infectious disease iceberg, in which the majority (80%) of WNV cases are asymptomatic, and approximately one in five cases develop WN fever, a flu-like illness [5]. Approximately one in every 150 cases progresses to a potentially fatal WN neurological disease (WNND), which can manifest as encephalitis, meningitis, or acute flaccid paralysis [5]. Approximately 10% of WNND cases will be fatal [6]. Although WNV was first detected in the U.S. in 1999, the first large outbreak occurred in 2002, with 2946 WNND cases and 284 deaths [6]. Beginning in 2002, the CDC has received reports of 386–2946 annual cases of WNND, causing 32–286 annual fatalities [6] (Figure 1). After large outbreaks in the U.S. in 2002 and 2003, there was a decrease in the number of WNND cases, but this was followed by another large outbreak in 2012. This periodic pattern of large outbreaks is typical of a mosquito-borne virus such as WNV [7]. Similarly, WNV is also endemic in parts of Europe, and 2018 marked the largest recorded WNV European outbreak in history, with >2000 reported cases and 181 deaths in 15 European countries [8]. Therefore, it can be anticipated that there will continue to be annual WNND cases along with large outbreaks that are difficult to predict as they depend on a multitude of environmental factors including temperature, precipitation, relative humidity, and landscape, as well as any other factors that may impact mosquito distribution, bird migration patterns, or urban development [9,10,11,12]. For these reasons, it is essential that WNV vaccine development continues to be pursued. 

## 2. Licensed Flavivirus Vaccines

While no human vaccine has been licensed for WNV, there are effective live, attenuated vaccines available for the related mosquito-borne viruses YFV, DENV, and JEV. Inactivated vaccines are also licensed for JEV and the tick-borne flaviviruses tick-borne encephalitis (TBEV) and Kyasanur forest disease (KFDV). The YFV live, attenuated vaccine strain 17D was empirically derived by serial passage, and incorporates 20 amino acid substitutions that differentiate the vaccine from the parental strain [13]. YFV 17D induces strong, typically life-long immunity with only one dose and has been associated with very few adverse events in the 82 years since its development [14]. In 2016, the first DENV vaccine was licensed as a tetravalent vaccine to protect against the four serotypes of DENV. The DENV vaccine (Dengvaxia™) is a chimeric live, attenuated vaccine that incorporates the nonstructural genes of YFV 17D along with the prM/E genes of DENV-1-4 [15]. The chimeric use of 17D, referred to as ChimeriVax technology, has been explored for use in development of multiple different flavivirus vaccines, including JEV (Imojev®). Although Dengvaxia™ has moderate efficacy after administration of three doses (0, 6, and 12 months), there are concerns over safety in vaccinees who are DENV seronegative at the time of immunization, and alternative candidate recombinant live, attenuated DENV vaccines are in clinical evaluation [16,17]. Finally, WNV and JEV are closely related both serologically and genetically, and licensed vaccines for JEV could be considered the most relevant to WNV vaccine development. There are multiple licensed JEV vaccines, including a live, attenuated vaccine strain SA14-14-2, formalin-inactivated JEV (using multiple virus strains), and the chimeric Imojev® vaccine combining the YFV 17D nonstructural genes with the prM/E genes from SA14-14-2 [18]. JE SA14-14-2 was empirically derived by serial passage in primary hamster kidney cells and harbors seventeen vaccine-specific amino acid differences from the parental strain SA14 [19,20]. SA14-14-2 has an excellent safety profile and can induce protective immunity for years following a single primary dose [21]. Some countries administer two booster doses of SA14-14-2, while others recommend only a single dose [22]. Likewise, the chimeric IMOJEV® vaccine is also safe and effective when administered with just a single dose, though pediatric boosters are recommended [23]. The inactivated Vero-cell-derived JEV vaccines also have an excellent safety profile but require two primary doses as well as a recommended booster dose [18,22]. In sum, flavivirus vaccines are licensed for five flaviviruses, three of which are mosquito-borne. Since there are multiple vaccines licensed against JEV, and since JEV and WNV share significant sequence homology (approximately 75% homology in coding sequence) [24], it is rational that a human WNV vaccine can also be developed.

## 3. Licensed WNV Veterinary Vaccines

WNV is not only pathogenic in humans, but is also a significant veterinary pathogen that has been associated with severe neurological disease and death in horses, wild mammalian and avian species, and a variety of animals found in zoological gardens. Several veterinary vaccines are commercially available, and their properties may help to inform work on human vaccine development. Currently, there are four WNV veterinary vaccines on the market: three that comprise whole inactivated virus (WN Innovator™, Vetera™ WNV, and Prestige®WNV) and one that is a live chimeric virus combining the WNV prM/E into a canarypox backbone (Recombitek™ Equine WNV) [25,26,27,28]. Although WNV veterinary vaccines are protective in horses [29,30,31], all four require two primary doses and an annual booster. Of note, although Recombitek™ WNV is a live virus, there is no evidence that canarypox-based vaccines are capable of replication in mammals [32], so canarypox vaccines do not elicit as strong immunity as many replicating live, attenuated vaccines.

## 4. Biological Properties of an Ideal Human WNV Vaccine

There are several properties that could be used to define an optimum human WNV vaccine candidate. WNV primarily causes severe disease in the elderly, and importantly, older age is associated with immunosenescence and a reduction of responsiveness to vaccination [33,34]. For a WNV vaccine to sufficiently protect the elderly, it likely needs to be capable of inducing a strong, long-lasting protective immune response. To date, neutralizing antibodies are the most reliable correlate of protection for flavivirus infections [35,36], and thus this is the primary measurement of vaccine efficacy. Besides neutralizing antibodies, there is a strong body of work indicating that WNV-specific T cells are also important components of protective immunity using mice and humans. Multiple groups have demonstrated that CD8^+^ T cells are needed to clear WNV infection in mice and are associated with enhanced protection after vaccination of mice with candidate WNV vaccines [37,38,39,40,41]. Alternatively, a recent study using a collaborative cross mouse model found that reduced regulatory T cells in the periphery and CD8^+^ T cells in the brain may be associated with protection from WNV neurological disease [42]. Although a balanced T cell response seems to be an essential component of WNV protective immunity in mice, additional studies are needed to determine if the same is true in humans, including identification of specific peptide targets associated with immunity in order to use them as a correlate of protection. However, it has been shown that a Chimerivax-WNV vaccine candidate induced WNV E protein-specific CD8^+^ T cell responses up to one year post vaccination in a clinical trial [43]. Since neutralizing antibody titers are used by the World Health Organization (WHO) as a correlate of protection for JEV [36], this will likely be the measurement for a WNV vaccine correlate of protection. It should be noted, however, that the definition of a correlate of protection is an immune marker (e.g., neutralizing antibodies) that is statistically associated with vaccine-induced protection, and this is different from a surrogate of protection, which is defined as an attribute on the direct causal pathway to vaccine-induced protection [44]. Currently, surrogates of WNV protection are not known, and prior to either vaccine efficacy studies in humans (i.e., a study undertaken under ideal clinical trial conditions) or vaccine effectiveness studies (i.e., a clinical study undertaken under field conditions), it is difficult to draw further conclusions as to what the appropriate correlate(s) of protection may be.

As a point of comparison for flavivirus vaccine efficacy, a single dose of the JE SA14-14-2 live, attenuated vaccine is capable of inducing neutralizing antibodies in children for at least five years [21,45]. SA14-14-2 is commonly administered to children as young as 8 months old in countries across Asia. Children typically have underdeveloped immune responses and thus require multiple doses of most vaccines, so the degree of protection described is significant. Furthermore, the YFV 17D vaccine is capable of inducing life-long protective immunity with a single dose in individuals 9 months and older [46]. These vaccines suggest that long-lived protective immunity can be induced by a live vaccine. Examination of the inactivated JE and TBE vaccines shows that intermediate levels of immunity can also be achieved using these vaccine platforms; however, multiple primary doses plus boosters are typically necessary. For instance, inactivated JE vaccines require two primary doses and a booster after 1–2 years, whereas inactivated TBEV vaccines require three primary doses and boosters every three to five years in order to maintain protection [47,48].

One limitation of immunogenicity studies is that most studies do not follow a standard protocol for measurement of neutralizing antibody titers, but instead, many different assays, endpoints, incubation times, and challenge viruses may be used. Therefore, it is not straightforward to directly compare the efficacy of different vaccine candidates. For JEV vaccines, the WHO has recommended that 50% plaque reduction neutralization (PRNT_50_) titers ≥10 be considered an immune correlate of protection in humans [36]. While the same PRNT_50_ titer is often considered protective in clinical trials of WNV vaccine candidates, some trials use alternative assays (e.g., PRNT_60_, reporter virus neutralization, microneutralization, etc.) or measure neutralization against a homologous vaccine strain rather than a virulent wild-type (WT) strain of WNV. Such differences in assay design inhibit comparison of vaccine candidates across studies, and this is a notable limitation when assessing which candidate(s) may be optimal.

Besides immunogenicity, another critical characteristic of a WNV vaccine is safety. It is critical that the risk of reversion of a candidate live, attenuated WNV vaccine is very low, especially considering that the vaccine needs to be safe for use in older populations with weakened immune responses. Correspondingly, the safest vaccination strategies utilize inactivated virus, non-replicating virus or subunit protein/DNA/RNA viral components, as the risk of reversion in these platforms is very low. Unfortunately, increased safety is often correlated with reduced efficacy, as non-replicating vaccines require multiple doses to induce a protective immune response. Although live, attenuated vaccines have the highest risk of reversion, it is possible to develop safe live vaccines, as has been demonstrated for YF, JE, measles, and varicella zoster viruses, for example. For JE SA14-14-2, which is most closely related to WNV, no serious adverse events or deaths have been directly associated with vaccination, even though over 300 million doses have been distributed [21]. An additional consideration for the safety of a live, attenuated WNV vaccine is the importance of reduced mosquito competence to prevent circulation of the vaccine strain in nature, which could result in unexpected alterations to the vaccine phenotype. Although JE SA14-14-2 does not decrease mosquito infection rates compared to the parental SA14 strain, vaccine isolated from mosquitoes was not virulent in mice and did not have any amino acid mutations following sequential passage between a mosquito vector and pig reservoir [20,49]. Moreover, YFV 17D and the chimeric JE vaccine IMOJEV® each have strongly reduced infection rates in both *Aedes* and *Culex spp*. mosquitoes, respectively [50,51].

In sum, a WNV vaccine should ideally induce robust long-term protective immunity, require a maximum of one booster dose, and if a live vaccine is developed it should show no evidence of reversion, and have a loss of mosquito competence.

## 5. Animal Models and Evaluation of Protective Immunity

Preclinical testing of candidate WNV vaccines must evaluate protective efficacy in animal models prior to moving to clinical evaluation. Due to the similarity of JEV and WNV, consideration of the animal models used for JEV vaccine testing can provide insight for WNV vaccine development. Mice are the most broadly used model to determine protective efficacy of JEV vaccines, as they generate neutralizing antibody responses upon vaccination and most mouse strains are highly susceptible to WT JEV, so peripheral challenge can adequately assess protection [36,52]. Many studies have demonstrated that JEV inactivated and live, attenuated vaccines in mice induce neutralizing antibody titers that correlate with survival from WT virus challenge [53,54,55,56,57], and the antibodies can provide protection against multiple JEV genotypes [56,57]. Significantly, a mouse model is used to evaluate vaccine potency by the WHO [58,59]. Similarly, for WNV vaccine development, neutralizing antibodies are typically investigated as the primary correlate of protection in both murine and human studies, and since WNV neuropathogenesis in mice is similar to that observed in humans [60], protection from murine neurological disease is predicted to correlate with protection in humans. However, this remains to be demonstrated in humans.

Alternative animals that have also been utilized for JEV and WNV vaccine efficacy studies are the hamster and non-human primate (NHP) models. The hamster model develops neutralizing antibodies that correlate with protection [61]. Although it is a sufficient model for immunogenicity studies, not all hamsters uniformly succumb to lethal wild-type WNV infection even though they develop high viremia (5–6 log_10_ PFU/mL) [61]; therefore, this model is often used to evaluate viremia or central nervous system persistence [62,63]. Some preclinical efficacy studies utilize NHPs, but this model requires large doses of WT challenge virus administered by the intracranial or intranasal routes to cause clinical disease [64]. Peripheral challenge of NHPs may be used to assess attenuation of viremia, but not that of neurological disease [65,66,67]. Since the mouse model has been most extensively studied for WNV vaccine candidates and it is an accepted model to evaluate protective immunity of JEV vaccines, it is probable that this animal model is adequate for preclinical evaluation of WNV vaccine efficacy.

## 6. Preclinical Studies of WNV Vaccines

Although WNV veterinary vaccines have been commercially available since WNV Innovator™ was licensed in 2003, development of a human vaccine has not been as rapid. There have been numerous WNV vaccine candidates proposed and evaluated in preclinical studies, including vaccine platforms based on live attenuated WNV, live chimeric WNV, DNA, protein, inactivated whole virus, and non-replicating sub-viral particles, as well as vaccination with platforms using heterologous, related viruses. Many of these vaccines have been compared previously in reviews published five to six years ago [68,69,70]. Since 2013–2014, when several WNV vaccine reviews were published, there have been additional advancements in WNV vaccine design. These include a replication-deficient Modified Vaccinia virus Ankara (MVA) to efficiently vector the WNV E protein (MVA-WNV) [71]. All mice vaccinated with 10^8^ PFU of MVA-WNV elicited detectable neutralizing antibodies, and antibody titers increased after a booster vaccination three weeks later [71]. Notably, mice vaccinated all survived challenge doses of 10^4^ PFU with both a lineage 1 and lineage 2 WT WNV strain, while mice vaccinated with the WT MVA vector all succumbed to the challenge [71]. Another WNV replication-deficient vaccine candidate focuses on a deletion of the nonstructural protein NS1. WNV-∆NS1 can be grown to high titers (10^8^ IU/mL), but the construct cannot replicate in naïve Vero cells nor in highly susceptible interferon αβ receptor deficient (IFNAR^-/-^) mice [72]. Moreover, a single dose of WNV-∆NS1 induced neutralizing antibodies and protected mice from a lethal challenge of WT WNV [72]. The high yield of the WNV-∆NS1 construct combined with the safety and efficacy in the mouse model make it a good candidate for further development as a vaccine platform.

## 7. Enhancing Vaccine Immunogenicity

Considering that there are already several WNV vaccine candidates that appear safe in phase I and II studies (see sections below on clinical trials), one strategy to aid in vaccine advancement is to boost the immune response to the vaccines currently available. For example, subunit vaccines often utilize aluminum-based salts (alum) as adjuvants, but other adjuvants have recently been licensed. For example, an alternative adjuvant, a toll-like receptor 4 (TLR4) agonist, is licensed for use in the human papilloma virus (HPV) vaccine Cervarix™ [73]. A TLR4 agonist was investigated for its ability to boost murine immune responses to the WNV subunit vaccine candidate, WN-80E [73]. When the TLR4 agonist was included as an adjuvant along with either alum or a stable oil-in-water emulsion, T cell responses, neutralizing antibody titers, and germinal center B cells increased compared to vaccination adjuvanted with alum only [73]. Although not yet tested in a clinical WNV vaccine trial, use of a TLR4 agonist for a licensed HPV vaccine and promising results in the WN-80E mouse studies suggest that this approach is worth further exploration.

In addition to TLR4 agonists, another novel agonist that has been explored is based on self-assembling peptide nanofibers. This adjuvant was evaluated with E protein domain III (EIII). Although WNV EIII is the primary target of neutralizing antibodies, EIII expressed alone does not induce robust immunity in mice when compared to other potential vaccine platforms [74,75]. WNV EIII was used to evaluate self-assembling peptides that form β-sheet rich nanofibers and enhance immune responses when combined with peptides or subunit proteins to generate a peptide hydrogel [76]. When EIII was combined with a peptide nanofiber, mice had increased survival after vaccination and increased EIII-specific antibody responses measured by ELISA compared to mice vaccinated with EIII + alum [76]. The use of peptide nanofibers warrants continued investigation as a next-generation vaccine adjuvant for WNV vaccines, as subunit protein vaccines typically need robust immune adjuvants to improve immunogenicity.

## 8. Vaccines in Clinical Evaluation

Several vaccine candidates have progressed from preclinical development into clinical evaluation. These include two recombinant live attenuated viruses, two whole inactivated viruses, one DNA, and one recombinant E protein. A summary of the vaccine characteristics can be found in Table 1 and the protective efficacy is summarized in Table 2.

## 9. Phase I Clinical Trials

One vaccine candidate that was tested in phase I clinical trials utilized plasmid DNA expressing WNV prM/E. The initial vaccine candidate utilized a cytomegalovirus (CMV) promoter to enhance gene expression (study name VRC 302), while a second clinical study modified the promoter to include an additional ‘R’ region (CMV/R promoter) derived from human T-cell leukemia virus type 1 (HTLV-1) to further increase expression (study name VRC 303) [77,78]. Both candidates were tested with a regimen of three primary doses of 4 mg DNA per dose by the intramuscular (i.m.) route. In the first trial, VRC 302, twelve healthy adults ages 19–44 completed the three dose schedule and all subjects had neutralizing antibody titers up to 32 weeks following vaccination [77]. In the second trial, VRC 303, two age groups were tested, with 15 subjects enrolled between the ages of 22 and 45, and 15 enrolled between the ages of 51 and 65 [78]. Twenty-eight of 29 subjects who received the vaccine developed neutralizing antibodies, and there was a trend toward more robust neutralizing antibody titers and T cell responses compared to the previous VRC 302 study [78]. Importantly, the elicited immune responses were comparable between the two age groups tested, and there was a trend toward better immunogenicity in the older age group. Notably, this group used a neutralization test that measured reduction of fluorescence of a reporter virus to identify the 50% effective concentration (EC_50_) of serum from vaccinees. This assay cannot be directly compared to classical PRNTs. The authors did report that both PRNT_50_ and reporter virus assays detected neutralizing antibodies in all vaccinated individuals in the VRC 302 study [77], but in the VRC 303 study, only the reporter virus assay was used [78]. Although this vaccine was well tolerated and was efficacious at least through 32 weeks, additional clinical trials have not been undertaken since the second phase I study that began in 2006.

Another vaccine candidate studied in phase I clinical trials was a recombinant E protein vaccine, termed WN-80E or HBV-002. The E protein used in this vaccine was produced in *Drosophila melanogaster* S2 cells and a transmembrane region was excluded to ensure solubility, therefore, the E protein is truncated and retains the N-terminal ectodomain comprising approximately 80% of the amino acids [79]. Three experimental doses (5, 15, or 50 µg) were tested with an alum adjuvant administered by the i.m. route. There were a total of 25 participants between the ages of 18 and 45. All subjects had neutralizing antibodies (measured by PRNT_50_) at two weeks post vaccination, with higher antibody titers resulting from the 15 and 50 µg doses; however, longer-term antibody titers were not reported [79]. This trial, which was initiated in 2008, is the last clinical study completed to evaluate WN-80E, so further details on immunogenicity and vaccine effectiveness in those >age 45 are lacking.

In addition to the DNA and protein vaccines described above, two chimeric live, attenuated vaccines have also been tested in phase I studies. The first uses a DENV backbone that combines the WNV prM/E with the DENV-4 nonstructural genes incorporating a 30 nucleotide deletion in the 3′ UTR (WN/DEN4∆30) [66]. The DENV4∆30 backbone has also been tested as a DENV vaccine candidate in phase III clinical trials (clinical trial NCT02406729). WN/DEN4∆30 has been tested in three WNV phase I clinical trials: the first two were studies of healthy individuals ages 18–50 whereas the third trial evaluated the vaccine in individuals ages 50–65. In the 18–50 year-old cohort, three potential regimens were evaluated: 10^3^ PFU (1 dose), 10^4^ PFU (1 dose), and 10^5^ PFU (2 doses, 6 months apart) given by the subcutaneous (s.c.) route [80]. The number of subjects analyzed for serology was 27, 28, and 21, respectively [80]. By day 180 post vaccination, seroconversion rates were 74% for the 10^3^ PFU inoculum, 75% for the 10^4^ PFU inoculum, and 55% for the 10^5^ PFU inoculum (after receiving one dose) [80]. Seroconversion rates for the 10^5^ PFU dose were boosted to 89% forty-two days after receiving the second dose [80]. Interestingly, viremia and PRNT_60_ titers were lower following a single dose of the 10^5^ PFU dose compared to either 10^3^ or 10^4^ PFU, but the authors note that this was observed for other flavivirus vaccines and may be due to differences in manufacturing of the different doses or in accumulation of quasispecies and defective interfering particles [80]. Overall, WN/DEN4∆30 was well tolerated and effective in the 18–50-year-old cohort. A third phase I trial completed between 2014–2016 focused on older adults ages 50–65 [81]. Twenty participants were given two doses of 10^4^ PFU of the vaccine six months apart [81]. Neutralizing antibodies measured by PRNT_50_ assays found that 95% of the participants seroconverted by day 90 post vaccination with a single dose [81]. Notably, this group tested neutralizing antibodies against two WT WNV strains (NY99 and WN02) plus the vaccine strain, so this ensured that protection would not be limited to only the homologous recombinant vaccine virus. Interestingly, neutralizing antibody titers were higher after a single dose of vaccine than they were after a second dose was administered. Importantly WN/DEN4∆30 also has reduced competence in both *Culex* and *Aedes spp*. mosquitoes, so the risk of vaccine transmission by mosquitoes is low. With the results currently available, WN/DEN4∆30 appears to be a good candidate for further evaluation in phase II clinical studies.

The most recent (2015–2016) vaccine to complete phase I clinical studies is HydroVax-001, a hydrogen peroxide-inactivated whole virus vaccine. Notably, all of the other vaccine candidates described previously (DNA, subunit protein, and chimeric live, attenuated) are based on the highly virulent WNV NY99 strain, but HydroVax-001 utilized the naturally attenuated WNV Kunjin strain. Twenty individuals aged 18–49 received 1 µg of HydroVax-001 by the i.m. route and twenty received 4 µg [82]. The vaccine was given in two doses, four weeks apart. The 1 µg dose did not induce neutralizing antibodies detectable by PRNT_50_, but there was evidence of WNV specific antibody responses in ELISA assays [82]. The 4 µg dose induced PRNT_50_ neutralizing antibody responses in up to 31% of the cohort, and this increased to up to 50% of the cohort when complement was added to the assay [82]. Overall, antibody responses peaked between 15 and 29 days following the second dose, and thereafter declined throughout the one year follow-up. Overall, HydroVax-001 was not strongly immunogenic and the vaccine would likely need to be improved prior to continued clinical studies. The authors noted that addition of a third dose may increase immunogenicity [82]. To further improve on the antigenic integrity and thus the immunogenicity of HydroVax-001, a second-generation, HydroVax-II, was developed using a new inactivation protocol [83]. The updated strategy used reduced concentrations of hydrogen peroxide combined with copper ions, the antiviral drug methisazone, as well as low concentrations of formaldehyde to reduce oxidative damage that results from hydrogen peroxide inactivation alone [83]. HydroVax-II was found to increase neutralizing epitope retention, neutralizing antibody titers, and survival in a mouse model when compared to the first-generation HyrdroVax-001 [83]. This novel method of vaccine inactivation may be beneficial to WNV vaccine development as well as vaccine development for other viruses.

## 10. Phase II Clinical Trials

A formalin-inactivated WNV vaccine is the only candidate that has been tested in phase I/II clinical trials simultaneously. A total of 320 participants aged 18 and older were enrolled and were assigned to receive two doses of vaccine 21 days apart followed by a booster dose after 180 days [84]. Vaccinees received either 1.25, 2.5, 5.0, or 10.0 µg of antigen with or without alum adjuvant by the i.m. route [84]. While all vaccine formulations elicited neutralizing antibody responses in the participants, the highest antibody titers resulted from vaccination with the 10 µg non-adjuvanted vaccine following the booster dose [84]. Notably, this study utilized a microneutralization assay as opposed to the traditional PRNT. For microneutralization, a reduction of cytopathic effect was measured rather than a reduction of virus plaques or foci [85], making the endpoint of this assay significantly different than that of PRNTs. There is limited published data describing this trial, and additional details on the immunogenicity of this formalin-inactivated vaccine are not available.

The only other candidate WNV vaccine that has been tested in phase II clinical trials is ChimeriVax-WN02, combining the WNV NY99 strain prM/E with the YFV 17D nonstructural genes. This is a second generation ChimeriVax-WN vaccine that includes three mutations in the E protein to enhance attenuation of this vaccine candidate [64]. Mutations of six E protein amino acid residues were evaluated in ChimeriVax-WN: residues 107, 138, 176, and 280, which are determinants of attenuation in JE SA14-14-2, and residues 316 and 440, which are in E residues implicated to be important to flavivirus biology [64]. Of the six mutations tested in ChimeriVax-WN, the three selected were at residues 107, 316, and 440 as they had the most profound effect on attenuation of neurovirulence in outbred mice [64]. ChimeriVax-WN02 was initially tested in a phase I trial and 41 subjects ages 18–40 all achieved seroconversion (measured by PRNT_50_) 21 days after a single dose of the vaccine, regardless of whether they received 10^3^ or 10^5^ PFU (route of administration not reported) [86]. The first of two phase II studies was completed using ChimeriVax-WN02 between 2005–2009, and 112 subjects between ages 18–40 and 96 subjects ≥ age 41 were enrolled [87]. The 18–40 age group received one of three vaccine doses by the s.c. route: 3.7 × 10^3^, 3.7 × 10^4^, or 3.7 × 10^5^ PFU, and those older than 41 received the 3.7 × 10^5^ PFU dose [87]. Each group only received a single dose of vaccine. All three age groups (18–40, ≥41–64, and ≥65) exhibited 96% seroconversion measured with PRNT_50_ assays by 28 days post vaccination. Interestingly, antibody titers generally waned after day 28, except for the 65 and older age group for which titers increased between study day 45 and month 6 [87]. Notably, the subjects ≥65 also had the highest antibody titers of the three age groups one year after vaccination. Considering the importance of older cohorts for WNV vaccine development, a second phase II study was completed from 2008–2009 focusing only on adults ≥age 50 [88]. There were 479 participants enrolled in this study and three different ChimeriVax-WN02 doses were tested: 4 × 10^3^, 4 × 10^4^, and 4 × 10^5^ PFU [88]. Regardless of the dose received, neutralizing antibody titers were similar between the groups and were not statistically different between age groups of 50–64 and ≥65 [88]. Overall, the seroconversion rate at 28 days post vaccination was 92% [88]. Unlike the WN/DEN4∆30 chimeric vaccine candidate, higher doses of ChimeriVax-WN02 corresponded with higher neutralizing antibody titers, though differences were not statistically significant. To summarize, of two phase II clinical trials that evaluated several age groups, including the elderly, ChimeriVax-WN02 was safe and effective, eliciting higher seroconversion rates after a single dose than those reported for other vaccine candidates; albeit it is not possible to compare the neutralization results between different candidates. Of note, the PRNT_50_ assays measured neutralizing antibodies against the chimeric vaccine strain; therefore, it is not clear whether neutralization would be similar when tested against WT WNV. Regardless, ChimeriVax-WN02 is the most extensively studied WNV vaccine candidate and it may be closest to licensure of the six candidate vaccines that have been evaluated in clinical trials.

## 11. Alternative Approaches for WNV Vaccine Development

Subsequent to the clinical trials, several other platforms have been investigated to protect the population from WNV disease. Since WNV is one of numerous mosquito-borne pathogens, there is interest in developing a vaccine that targets mosquito saliva. Ideally, a vaccine could be generated against proteins in mosquito saliva that are associated with virus transmission, and thus vaccinated individuals would be protected from multiple mosquito-borne pathogens instead of targeting each pathogen individually. As with any vaccination strategy, a mosquito-targeted vaccine has its own list of benefits and limitations, which have recently been published in a comprehensive review article [89]. This concept is being investigated in humans and the first phase I clinical trial was completed between 2017 and 2018, although the results have not yet been reported [90].

## 12. Challenges of Licensing and Marketing a WNV Vaccine

A practical concern for WNV vaccine development is undertaking phase III clinical trials. The WNV season is relatively short from May/June-September/October each year. In addition, WNV outbreaks tend be restricted and vary in geographical area each year, so the time it takes to identify an outbreak poses a challenge, as diagnostics for virus-infected mosquito pools or virus-infected birds/humans are often not done in real-time. Furthermore, once the outbreak has been identified it takes time to set up a phase III clinical trial, including local Investigational Review Board approval. Although phase III studies were successfully undertaken for inactivated JEV vaccines [91], there are concerns about high cost and the feasibility of identifying a suitable target population and endemic region for WNV efficacy studies in a short period of time. As such, there are practical hurdles to be overcome for a phase III trial to be undertaken.

In addition, marketing of a new vaccine would be an expensive endeavor, so it is important that a vaccine be cost effective. The cost of direct and indirect medical care of individuals hospitalized for WNV in the United States was estimated to be on average $56 million dollars per year [92], demonstrating the substantial economic impact that WNV continues to cause. Using mathematical modeling, it has been suggested that a vaccination program in the United States targeting adults ≥50 years old would be more cost effective than a universal vaccination program [93]. In particular, the 60-year-cohort had a mean cost per neuroinvasive disease case prevented of $664,000. However, the model used couldn’t take into account a specific vaccine cost, vaccine efficacy, or duration of protection, but they did report that, not surprisingly, a single dose vaccination would be more cost effective than vaccines requiring multiple doses [93]. Recognizing that the elderly are at the greatest risk for severe WNV disease, it is significant that in clinical studies of the live vaccine candidates WN/DEN4∆30 and ChimeriVax-WN02 there have been no reports of any serious adverse events in any age group, including the adults older than 50 [80,81,87]. The exhibited safety of live vaccines tested so far is encouraging that a safe single dose WNV vaccine can be developed.

## 13. Conclusions

Since WNV emerged into the U.S. twenty years ago, there has been excellent progress toward the development of a vaccine. Fortunately, there are four licensed veterinary vaccines to protect horses. While veterinary vaccines have provided insights for the process of human vaccine development, no human vaccines have yet been tested beyond phase II clinical trials. The most extensively studied candidate, ChimeriVax-WN02, proved to be safe and effective in multiple age groups, including the most susceptible elderly population, and therefore, this vaccine has potential for continued development. As the most recent ChimeriVax-WN02 clinical trial took place approximately ten years ago, many groups are still working to develop and refine other vaccination strategies. Regardless of the development of seemingly safe and effective WNV vaccination strategies, undertaking phase III efficacy studies and marketing of a vaccine are practical challenges that remain. Emphasis should be on safe and strongly immunogenic vaccine candidates, as a single dose vaccine will be most likely to achieve licensure as it is most cost effective. To date, the two recombinant live attenuated vaccines in clinical evaluation have generated the best data as candidate vaccines for use in humans, despite that immunogenicity data cannot be directly compared since neutralization assays often differ between studies. As expected, a live vaccine may be the best platform as they are often strongly immunogenic with only a single dose, but safety is a major issue for a live attenuated WNV vaccine, especially considering that the target population is likely to be older adults. Many novel techniques are being discovered to boost immunogenicity from other vaccine platforms as well, and to date there has been no RNA vaccine candidate evaluated as a WNV vaccine [94]. In summary, twenty years of WNV vaccine development has contributed greatly to the fields of flavivirus virology and viral vaccine development, and the characteristics of available vaccine candidates are encouraging that a safe and effective WNV vaccine for humans can be licensed.

## Figures and Tables

**Table 1:** Summary of WNV vaccine candidates that have been studied in clinical trials.

Vaccine	Developer	Vaccine Type	WNV Strain	Clinical Trial (Onset)	Clinical Trial Number	Dose and Route	Dosing Series
^1^ VRC 302 ^2^ VRC 303	NIAID Vaccine Research Center	prM/E DNA with ^1^ CMV or ^2^ CMV/R promoter	NY99	Phase I (2006)	^1^ NCT00106769 ^2^ NCT00300417	4 mg i.m.	Three doses four weeks apart
WN-80E	Hawaii Biotech	Recombinant, truncated E protein	NY99	Phase I (2008)	NCT00707642	5, 15, or 50 µg i.m.	Three doses four weeks apart
WN/DEN4∆30	NIAID Division of Intramural Research	Chimeric, live virus with WNV prM/E and DENV-4 nonstrucutral genes with a 30 nt deletion	NY99	Phase I (2004) Phase I (2007) Phase I (2014)	NCT00094718 NCT00537147 NCT02186626	10^3^, 10^4^, or 10^5^ PFU s.c.	* One or two doses
HydroVax-001	Najit Technologies	Hydrogen peroxide-inactivated whole virus	Kunjin	Phase I (2015)	NCT02337868	1 or 4 µg i.m.	Two doses four weeks apart
Formalin-inactivated WNV	Nanotherapeutics Inc.	Formalin-inactivated whole virus	NY99	Phase I/II	none	1.25, 2.5, 5.0, or 10.0 µg i.m.	Two doses 21 days apart plus booster dose on day 180
ChimeriVax-WN02	Sanofi Pasteur	Chimeric, live virus with WNV prM/E and YFV 17D nonstrucutral genes with three site-directed mutations in the E protein	NY99	Phase 1 Phase II (2005) Phase II (2008)	none NCT00442169 NCT00746798	10^3^, 10^4^, or 10^5^ PFU s.c.	One dose

^1^ Corresponds to the study NCT00106769 using the CMV promoter. ^2^ Corresponds to the study NCT00300417 using the modified CMV/R promoter. * Although WN/DEN4∆30 was tested in a two-dose regimen, one dose was found to provide better neutralizing antibody responses in older adults NIAID = National Institute of Allergy and Infectious Diseases, CMV = cytomegalovirus, nt = nucleotide, i.m. = intramuscular, s.c. = subcutaneous.

**Table 2:** WNV vaccine candidates use different assays to determine protection by seroconversion.

Vaccine	Neutralization Assay	Challenge Virus	Seroconversion Definition	Seroconversion Rate (Time Post Vaccination)	Neutralization Titer Range ^#^	Age Group
**VRC 303**	Neutralization of reporter virus particles (reduction of fluorescence)	Reporter virus with NY99 structural components	above limit of detection	97% (12 weeks)	~ 20–10,000	22–65
**WN-80E**	PRNT_50_	unknown	≥ 1:10 dilution	100% (2 weeks)	~ 50–100	18–45
**WN/DEN4∆30**	^(a)^ PRNT_60_ ^(b)^ PRNT_50_	NY99, WN02, WN/DEN4∆30	^(a)^ ≥ 4-fold increase from baseline ^(b)^ ≥ 1:10 dilution	*^,(a)^ 75% for NY99 (180 days) ^(b)^ 95% for each challenge virus (90 days)	^(a)^ ≤ 5–232 ^(b)^ 38–134	^(a)^ 18–50 ^(b)^ 50–65
**HydroVax-001**	PRNT_50_	unknown	≥ 1:20 dilution	31% (15 days)	^∞^ 9.8	18–49
**Formalin-inactivated WNV**	Microneutraliztion (reduction of CPE)	unknown	n.d.	n.d.	^∞^ ~ 140	≥ 18
**ChimeriVax-WN02**	PRNT_50_	ChimeriVax-WN02	≥ 4-fold increase from baseline	^(c)^ 96% (28 days) ^(d)^ > 92% (28 days)	^∞,(c)^ 3309 ^∞,(d)^ 674	^(c)^ 18–80 ^(d)^ 50–88

Seroconversion rates and neutralization titers are representative of the vaccine regimens that gave the strongest antibody responses. * Seroconversion rates reported for WN/DEN4∆30 are following a single 10^4^ PFU dose as this was the most immunogenic single dose tested and it was used in both age groups reported. ^#^ Neutralization titer range is shown as the reciprocal serum dilution that satisfied the endpoint of the given assay at the time post vaccination listed in the seroconversion rate column. ^∞^ A range was not reported, so the value represents the geometric mean titer. ^(a)^ Corresponds to the studies NCT00094718 and NCT00537147. ^(b)^ Corresponds to the study NCT02186626. ^(c)^ Corresponds to the study NCT00442169. The neutralization titer reported is from the 105 PFU dose as this elicited the strongest antibody response of three doses tested. ^(d)^ Corresponds to the study NCT00746798. The neutralization titer reported is from the 105 PFU dose. EC = effective concentration, PRNT = plaque reduction neutralization test, CPE = cytopathic effect, n.d. = no data.

## References

[1] Kuno G., Chang G.J., Tsuchiya K.R., Karabatsos N., Cropp C.B. (1998). Phylogeny of the Genus Flavivirus. J. Virol..

[2] Smithburn K.C., Hughes T.P., Burke A.W., Paul J.H. (1940). A neurotropic virus isolated from the blood of a native of uganda. Am. J. Trop. Med. Hyg..

[3] Chancey C., Grinev A., Volkova E., Rios M. (2015). The global ecology and epidemiology of west nile virus. Biomed Res. Int..

[4] West Nile Virus. https://www.cdc.gov/westnile/index.html.

[5] West Nile Virus—Symptoms, Diagnosis, & Treatment. https://www.cdc.gov/westnile/symptoms/index.html.

[6] West Nile Virus—Statistics and Maps. https://www.cdc.gov/westnile/statsmaps/index.html.

[7] Rosenberg R., Lindsey N.P., Fischer M., Gregory C.J., Hinckley A.F., Mead P.S., Paz-Bailey G., Waterman S.H., Drexler N.A., Kersh G.J. (2018). Vital signs: Trends in reported vectorborne disease cases—United States and Territories, 2004–2016. Morb. Mortal. Wkly. Rep..

[8] Barrett A. (2018). West Nile in Europe: An increasing public health problem. J. Travel Med..

[9] Paz S., Semenza J.C. (2013). Environmental Drivers of West Nile Fever Epidemiology in Europe and Western Asia—A Review. Int. J. Environ. Res. Public Health.

[10] Brown H.E., Childs J.E., Diuk-Wasser M.A., Fish D. (2008). Ecologic Factors Associated with West Nile Virus Transmission, Northeastern United States. Emerg. Infect. Dis..

[11] Marcantonio M., Rizzoli A., Metz M., Rosa R., Marini G., Chadwick E., Neteler M. (2015). Identifying the Environmental Conditions Favouring West Nile Virus Outbreaks in Europe. PLoS ONE.

[12] Hess A., Davis J.K., Wimberly M.C. (2018). Identifying Environmental Risk Factors and Mapping the Distribution of West Nile Virus in an Endemic Region of North America. GeoHealth.

[13] Beck A.S., Barrett A.D.T. (2015). Current status and future prospects of yellow fever vaccines. Expert Rev. Vaccines.

[14] Martins R.D.M., Leal M.D.L.F., Homma A. (2015). Serious adverse events associated with yellow fever vaccine. Hum. Vaccines Immunother..

[15] Guirakhoo F., Pugachev K., Zhang Z., Myers G., Levenbook I., Draper K., Lang J., Ocran S., Mitchell F., Parsons M. (2004). Safety and Efficacy of Chimeric Yellow Fever-Dengue Virus Tetravalent Vaccine Formulations in Nonhuman Primates. J. Virol..

[16] Clapham H.E., Wills B.A. (2018). Implementing a dengue vaccination programme—Who, where and how?. Trans. R. Soc. Trop. Med. Hyg..

[17] Beth D.K., Anna P.D., Kristen K.P., Marya P.C., Cecilia M.T., Palmtama L.G., Noreen H., Sean A.D., Dan E., Adrienne P.J. (2015). Robust and Balanced Immune Responses to All 4 Dengue Virus Serotypes Following Administration of a Single Dose of a Live Attenuated Tetravalent Dengue Vaccine to Healthy, Flavivirus-Naive Adults. J. Infect. Dis..

[18] Chen H.L., Chang J.K., Tang R. (2015). Bin Current recommendations for the Japanese encephalitis vaccine. J. Chin. Med. Assoc..

[19] Ni H.L., Chang G.J.J., Xie H., Trent D.W., Barrett A.D.T. (1995). Molecular basis of attenuation of neurovirulence of wild- type Japanese encephalitis virus strain SA14. J. Gen. Virol..

[20] Yu Y. (2010). Phenotypic and genotypic characteristics of Japanese encephalitis attenuated live vaccine virus SA14-14-2 and their stabilities. Vaccine.

[21] Ginsburg A.S., Meghani A., Halstead S.B., Yaich M., Sarah A., Meghani A., Halstead S.B., Yaich M. (2017). Use of the live attenuated Japanese Encephalitis vaccine SA14–14–2 in children: A review of safety and tolerability studies. Hum. Vaccines Immunother..

[22] Japanese Encephalitis Virus—Vaccines. https://www.who.int/ith/vaccines/japanese_encephalitis/en/.

[23] Chokephaibulkit K., Houillon G., Feroldi E., Bouckenooghe A. (2016). Safety and immunogenicity of a live attenuated Japanese encephalitis chimeric virus vaccine (IMOJEV^®^) in children. Expert Rev. Vaccines.

[24] Tanaka M., Haishi S., Aira Y., Kurihara S., Morita K., Igarashi A. (1991). Homology among Eleven Flavivirus by Comparative Nucleotide Sequence of Geomic RNAs and Deduced Amino Acid Sequences of Viral Proteins. Trop. Med..

[25] Ng T., Hathaway D., Jennings N., Champ D., Chiang Y.W., Chu H.J. (2003). Equine vaccine for West Nile virus. Dev. Biol..

[26] Vetera WNV. https://www.bi-vetmedica.com/species/equine/products/vetera_vaccines/Vetera_WNV.html.

[27] El Garch H., Minke J.M., Rehder J., Richard S., Edlund Toulemonde C., Dinic S., Andreoni C., Audonnet J.C., Nordgren R., Juillard V. (2008). A West Nile virus (WNV) recombinant canarypox virus vaccine elicits WNV-specific neutralizing antibodies and cell-mediated immune responses in the horse. Vet. Immunol. Immunopathol..

[28] Prestige WNV. https://merckusa.cvpservice.com/product/basic/view/1047544.

[29] Schuler L., Khaitsa M., Dyer N., Stoltenow C. (2004). Evaluation of an outbreak of West Nile virus infection in horses: 569 cases (2002). J. Am. Vet. Med. Assoc..

[30] Gardner I., Wong S., Ferraro G., Balasuriya U., Hullinger P., Wilson W., Shi P., MacLachlan N. (2007). Incidence and effects of West Nile virus infection in vaccinated and unvaccinated horses in California. Vet. Res..

[31] Angenvoort J., Brault A.C., Bowen R.A., Groschup M.H. (2013). West Nile viral infection of equids. Vet. Microbiol..

[32] Plotkin S.A., Cadoz M., Meignier B., Meric C., Leroy O., Excler J.L., Tartaglia J., Paoletti E., Gonczol E., Chappuis G. (1995). The safety and use of canarypox vectored vaccines. Dev. Biol. Stand..

[33] Weinberger B. (2012). Vaccines for the elderly. Clin. Microbiol. Infect..

[34] Amanna I.J. (2013). Balancing the Efficacy and Safety of Vaccines in the Elderly. Open Longev. Sci..

[35] Markoff L. (2000). Points to consider in the development of a surrogate for efficacy of novel Japanese encephalitis virus vaccines. Vaccine.

[36] Hombach J., Solomon T., Kurane I., Jacobson J., Wood D. (2005). Report on a WHO consultation on immunological endpoints for evaluation of new Japanese encephalitis vaccines, WHO, Geneva, 2-3 September, 2004. Vaccine.

[37] Engle M.J., Diamond M.S. (2003). Antibody prophylaxis and therapy against West Nile virus infection in wild-type and immunodeficient mice. J. Virol..

[38] Shrestha B., Diamond M.S. (2004). Role of CD8+ T cells in control of West Nile virus infection. J. Virol..

[39] Brien J.D., Uhrlaub J.L., Hirsh A., Wiley C.A., Nikolich-Zugich J. (2009). Key role of T cell defects in age-related vulnerability to West Nile virus. J. Exp. Med..

[40] Shrestha B., Ng T., Chu H., Noll M., Diamond M. (2008). The relative contribution of antibody and CD8+ T cells to vaccine immunity against West Nile encephalitis virus. Vaccine.

[41] Uhrlaub J.L., Brien J.D., Widman D.G., Mason P.W., Nikolich-zugich J. (2011). Repeated in vivo stimulation of T and B cell responses in old mice generates protective immunity against lethal West Nile virus encephalitis. J. Immunol..

[42] Graham J.B., Swarts J.L., Thomas S., Voss K.M., Sekine A., Green R., Ireton R.C., Gale M., Lund J.M. (2019). Immune Correlates of Protection from West Nile Virus Neuroinvasion and Disease. J. Infect. Dis..

[43] Smith H.L., Monath T.P., Pazoles P., Rothman A.L., Casey D.M., Terajima M., Ennis F.A., Guirakhoo F., Green S. (2011). Development of antigen-specific memory CD8+ T cells following live-attenuated chimeric West Nile virus vaccination. J. Infect. Dis..

[44] Correlates of Vaccine-Induced Protection: Methods and Implications. https://apps.who.int/iris/bitstream/handle/10665/84288/WHO_IVB_13.01_eng.pdf;sequence=1.

[45] Sohn Y.M., Tandan J.B., Yoksan S., Ji M., Ohrr H. (2008). A 5-year follow-up of antibody response in children vaccinated with single dose of live attenuated SA14-14-2 Japanese encephalitis vaccine: Immunogenicity and anamnestic responses. Vaccine.

[46] Yellow Fever Vaccine. https://www.cdc.gov/yellowfever/vaccine/index.html.

[47] Dubischar-Kastner K., Eder S., Buerger V., Gartner-Woelfl G., Kaltenboeck A., Schuller E., Tauber E., Klade C. (2010). Long-term immunity and immune response to a booster dose following vaccination with the inactivated Japanese encephalitis vaccine IXIARO^®^, IC51. Vaccine.

[48] Aerssens A., Cochez C., Niedrig M., Heyman P., Kühlmann-Rabens I., Soentjens P. (2016). Analysis of delayed TBE-vaccine booster after primary vaccination. J. Travel Med..

[49] Liu X., Jia L., Nie K., Zhao D., Na R., Xu H., Cheng G., Wang J., Yu Y., Li Y. (2019). Evaluation of environment safety of a Japanese encephalitis live attenuated vaccine. Biologicals.

[50] Bhatt T.R., Crabtree M.B., Guirakhoo F., Monath T.P., Miller B.R. (2000). Growth Characteristics of the Chimeric Japanese Encephalitis Virus Vaccine Candidate, ChimeriVax-JE (YF/JE SA14-14-2), in Culex Tritaeniorhychus, Aedes Albopictus, and Aedes Aegypti Mosquitoes. Am. J. Trop. Med. Hyg..

[51] Danet L., Beauclair G., Berthet M., Moratorio G., Gracias S., Tangy F., Choumet V., Jouvenet N. (2019). Midgut barriers prevent the replication and dissemination of the yellow fever vaccine in Aedes aegypti. PLoS Negl. Trop. Dis..

[52] Hills S., Walter E., Atmar R., Fischer M. (2019). Japanese encephalitis vaccine: Recommendations of the Advisory Committee on Immunization Practices. MMWR Recomm. Rep..

[53] Hammon W.M., Sather G.E. (1973). Passive immunity for arbovirus infection: I. Artificially Induced Prophylaxis in man and mouse for Japanese (B) encephalitis. Am. J. Trop. Med. Hyg..

[54] Lubiniecki A., Cypress R., WM H. (1973). Passive immunity for arbovirus infection. II. Quantitative aspects of naturally and artificially acquired protection in mice for Japanese (B) encephalitis virus. Am. J. Trop. Med. Hyg..

[55] Konishi E., Yamaoka M., Khin-Sane-Win, Kurane I., Takada K., Mason P. (1999). The anamnestic neutralizing antibody response is critical for protection of mice from challenge following vaccination with a plasmid encoding the Japanese encephalitis virus premembrane and envelope genes. J. Virol..

[56] Beasley D.W.C., Li L., Suderman M.T., Guirakhoo F., Trent D.W., Monath T.P., Shope R.E., Barrett A.D.T. (2004). Protection against Japanese encephalitis virus strains representing four genotypes by passive transfer of sera raised against ChimeriVax^TM^-JE experimental vaccine. Vaccine.

[57] Van Gessel Y., Klade C.S., Putnak R., Formica A., Krasaesub S., Spruth M., Cena B., Tungtaeng A., Gettayacamin M., Dewasthaly S. (2011). Correlation of protection against Japanese encephalitis virus and JE vaccine (IXIARO ^®^) induced neutralizing antibody titers. Vaccine.

[58] Recommendations for Japanese Encephalitis Vaccine (Inactivated) for Human Use (Revised 2007). https://www.who.int/biologicals/vaccines/Annex_1_WHO_TRS_963.pdf?ua=1.

[59] Recommendations to Assure the Quality, Safety and Efficacy of Japanese Encephalitis Vaccines (live, attenuated) for human use. https://www.who.int/biologicals/vaccines/JE-Recommendations_TRS_980_Annex_7.pdf?ua=1.

[60] Kimura T., Sasaki M., Okumura M., Kim E., Sawa H. (2010). Flavivirus encephalitis: Pathological aspects of mouse and other animal models. Vet. Pathol..

[61] Siirin M.T., Travassos da Rosa A.P.A., Newman P., Weeks-Levy C., Coller B.-A., Xiao S.-Y., Lieberman M.M., Watts D.M. (2008). Evaluation of the Efficacy of a Recombinant Subunit West Nile Vaccine in Syrian Golden Hamsters. Am. J. Trop. Med. Hyg..

[62] Xiao S.Y., Guzman H., Zhang H., Travassos Da Rosa A.P.A., Tesh R.B. (2001). West Nile virus infection in the golden hamster (Mesocricetus auratus): A model for west Nile encephalitis. Emerg. Infect. Dis..

[63] Tesh R.B., Siirin M., Guzman H., Travassos da Rosa A.P.A., Wu X., Duan T., Lei H., Nunes M.R., Xiao S. (2005). Persistent West Nile Virus Infection in the Golden Hamster: Studies on Its Mechanism and Possible Implications for Other Flavivirus Infections. J. Infect. Dis..

[64] Arroyo J., Miller C., Catalan J., Myers G.A., Ratterree M.S., Trent D.W., Monath T.P. (2004). ChimeriVax-West Nile Virus Live-Attenuated Vaccine: Preclinical Evaluation of Safety, Immunogenicity, and Efficacy. J. Virol..

[65] Lieberman M.M., Nerurkar V.R., Luo H., Cropp B., Carrion R., De La Garza M., Coller B.A., Clements D., Ogata S., Wong T. (2009). Immunogenicity and protective efficacy of a recombinant subunit West Nile Virus vaccine in rhesus monkeys. Clin. Vaccine Immunol..

[66] Pletnev A.G., Swayne D.E., Speicher J., Rumyantsev A.A., Murphy B.R. (2006). Chimeric West Nile/dengue virus vaccine candidate: Preclinical evaluation in mice, geese and monkeys for safety and immunogenicity. Vaccine.

[67] Poore E.A., Slifka D.K., Thomas A., Quintel B.K., Torrey L.L., Slifka A.M., Justin M., Dubois M.E., Johnson L.P., Diamond M.S. (2017). Pre-clinical development of a hydrogen peroxide-inactivated West Nile virus vaccine. Vaccine.

[68] Brandler S., Tangy F. (2013). Vaccines in Development against West Nile Virus. Viruses.

[69] Iyer A.V., Kousoulas K.G. (2013). A Review of Vaccine Approaches for West Nile Virus. Int. J. Environ. Res. Public Health.

[70] Amanna I.J., Slifka M.K. (2014). Current Trends in West Nile Virus Vaccine Development. Expert Rev. Vaccines.

[71] Volz A., Lim S., Kaserer M., Lülf A., Marr L., Jany S., Deeg C.A., Pijlman G.P., Koraka P., Osterhaus A.D.M.E. (2016). Immunogenicity and protective efficacy of recombinant Modified Vaccinia virus Ankara candidate vaccines delivering West Nile virus envelope antigens. Vaccine.

[72] Li N., Zhang Y.-N., Deng C.-L., Shi P.-Y., Yuan Z.-M., Zhang B. (2019). Replication-defective West Nile virus with NS1 deletion as a new vaccine platform for flavivirus. J. Virol..

[73] Van Hoeven N., Joshi S.W., Nana G.I., Bosco A., Fox C., Bowen R.A., Clements D.E., Martyak T., Parks E., Baldwin S. (2016). A Novel Synthetic TLR-4 Agonist Adjuvant Increases the Protective Response to a Clinical-Stage West Nile Virus Vaccine Antigen in Multiple Formulations. PLoS ONE.

[74] Chu J.J., Chiang C.S., Ng M., Alerts E. (2007). Immunization of Flavivirus West Nile Recombinant Envelope Domain III Protein Induced Specific Immune Response and Protection against West Nile Virus Infection. J. Immunol..

[75] Zlatkovic J., Stiasny K., Heinz F.X. (2011). Immunodominance and Functional Activities of Antibody Responses to Inactivated West Nile Virus and Recombinant Subunit Vaccines in Mice. J. Virol..

[76] Friedrich B.M., Beasley D.W.C., Rudra J.S. (2016). Supramolecular peptide hydrogel adjuvanted subunit vaccine elicits protective antibody responses against West Nile virus. Vaccine.

[77] Martin J.E., Pierson T.C., Hubka S., Rucker S., Gordon I.J., Enama M.E., Andrews C.A., Xu Q., Davis B.S., Nason M.C. (2007). A West Nile virus DNA vaccine induces neutralizing antibody in healthy adults during a phase 1 clinical trial. J. Infect. Dis..

[78] Ledgerwood J.E., Pierson T.C., Hubka S.A., Desai N., Rucker S., Gordon I.J., Enama E., Nelson S., Nason M., Gu W. (2011). A West Nile Virus DNA Vaccine Utilizing a Modified Promoter Induces Neutralizing Antibody in Younger and Older Healthy Adults in a Phase I Clinical Trial. J. Infect. Dis..

[79] Coller I.B., Pai V., Weeks-levy C.L., Ogata S.A. (2017). Recombinant Subunit West Nile Virus Vaccine for Protection of Human Subjects. U.S. Patent.

[80] Durbin A.P., Wright P.F., Cox A., Kagucia W., Elwood D., Henderson S., Wanionek K., Speicher J., Whitehead S.S., Pletnev A.G. (2013). The live attenuated chimeric vaccine rWN/DEN4 30 is well-tolerated and immunogenic in healthy flavivirus-naïve adult volunteers. Vaccine.

[81] Pierce K.K., Whitehead S.S., Kirkpatrick B.D., Grier P.L. (2017). A Live Attenuated Chimeric West Nile Virus Vaccine, rWN/DEN4Δ30, Is Well Tolerated and Immunogenic in Flavivirus-Naive Older Adult Volunteers. J. Infect. Dis..

[82] Woods C.W., Sanchez A.M., Swamy G.K., Mcclain M.T., Harrington L., Freeman D., Poore E.A., Slifka D.K., Poer D.E., Amanna I.J. (2019). An observer blinded, randomized, placebo-controlled, phase I dose escalation trial to evaluate the safety and immunogenicity of an inactivated West Nile virus Vaccine, HydroVax-001, in healthy adults. Vaccine.

[83] Quintel B.K., Thomas A., Poer D.E., Slifka M.K., Amanna I.J. (2019). Advanced oxidation technology for the development of a next-generation inactivated West Nile virus vaccine. Vaccine.

[84] Barrett P.N., Terpening S.J., Snow D., Cobb R.R., Kistner O. (2017). Vero cell technology for rapid development of inactivated whole virus vaccines for emerging viral diseases. Expert Rev. Vaccines.

[85] Orlinger K.K., Holzer G.W., Schwaiger J., Mayrhofer J., Schmid K., Kistner O., Noel Barrett P., Falkner F.G. (2010). An inactivated West Nile Virus vaccine derived from a chemically synthesized cDNA system. Vaccine.

[86] Monath T.P., Liu J., Kanesa-thasan N., Myers G.A., Nichols R., Deary A., Mccarthy K., Johnson C., Shin S., Arroyo J. (2006). A live, attenuated recombinant West Nile virus vaccine. Proc. Natl. Acad. Sci. USA.

[87] Biedenbender R., Bevilacqua J., Gregg A.M., Watson M., Dayan G. (2011). Phase II, randomized, double-blind, placebo-controlled, multicenter study to investigate the immunogenicity and safety of a West Nile virus vaccine in healthy adults. J. Infect. Dis..

[88] Dayan G.H., Bevilacqua J., Coleman D., Buldo A., Risi G. (2012). Phase II, dose ranging study of the safety and immunogenicity of single dose West Nile vaccine in healthy adults ≥ 50 years of age. Vaccine.

[89] Manning J.E., Morens D.M., Kamhawi S., Valenzuela J.G., Memoli M. (2018). Mosquito Saliva. The Hope for a Universal Arbovirus Vaccine?. J. Infect. Dis..

[90] Study in Healthy Volunteers to Evaluate the Safety and Immunogenicity of AGS-v, a Universal Mosquito-Borne Disease Vaccine. https://clinicaltrials.gov/ct2/show/study/NCT03055000.

[91] Hoke C.H., Nisalak A., Sangawhipa N., Jatanasen S., Laorakapongse T., Innis B.L. (1988). Protection against Japanese Encephalitis by Inactivated Vaccines. N. Engl. J. Med..

[92] Staples J.E., Shankar M.B., Sejvar J.J., Meltzer M.I., Fischer M. (2014). Initial and Long-Term Costs of Patients Hospitalized with West Nile Virus Disease. Am. J. Trop. Med. Hyg..

[93] Shankar M.B., Staples J.E., Meltzer M.I., Fischer M. (2017). Cost effectiveness of a targeted age-based West Nile virus vaccination program. Vaccine.

[94] Pardi N., Hogan M.J., Porter F.W., Weissman D. (2018). mRNA vaccines-a new era in vaccinology. Nat. Rev. Drug Discov..

